# Enhancing meat preservation with eco-friendly, antibiotic-free yeast-encapsulated essential oils

**DOI:** 10.1007/s11274-026-05213-4

**Published:** 2026-08-27

**Authors:** Behnaz Azimzadeh, Muiz O. Akinyemi, Bukola A. Onarinde

**Affiliations:** 1 School of Agri-Food Technology and Manufacturing, University of Lincoln, Holbeach, PE12 7FJ UK; 2 Leeds Institute of Health Sciences, University of Leeds, Leeds, LS2 9LN UK; 3 Unit for Environmental Sciences and Management, North-West University, Potchefstroom, South Africa

**Keywords:** Natural preservative, Food-grade carrier, Food preservation, Natural antimicrobial, Food safety

## Abstract

The application of essential oils as biopreservatives is hindered by their high volatility, chemical instability, and potential sensory impact. This study presents an eco-friendly preservation strategy by encapsulating the most potent antimicrobial EOs within *Saccharomyces cerevisiae* cells. These oils were subsequently encapsulated into autolyzed yeast cells, a process optimized at 45 °C to maximize cell integrity and oil loading. Encapsulation efficiency varied with EO chemistry, being highest for cinnamon (eugenol-rich) and lowest for rosemary (camphor-rich). In vitro assays showed encapsulation differentially modulated efficacy. It reduced the minimum inhibitory concentration (MIC) of cinnamon EO by 50% (from 6.25 to 3.12 µL, p < 0.05) while increasing the MIC for thyme and oregano. Importantly, in a real-world ham model, yeast-encapsulated EOs applied at their MIC achieved a superior, sustained bactericidal effect against *E. coli* over a six-day storage period. Encapsulated thyme and cinnamon reduced bacterial survival to below detectable limits within 24 h under specific packaging, outperforming free EO treatments (p < 0.001). These findings demonstrate that yeast microencapsulation successfully overcomes the key limitations of EOs, transforming them into stable, effective, and natural preservatives. The study provides proof-of-concept for *S. cerevisiae* as a GRAS, biodegradable delivery system, offering a promising antibiotic-free approach to enhance the safety and shelf-life of meat products.

**Supplementary Information:**

The online version contains supplementary material available at https://doi.org/10.1007/s11274-026-05213-4.

## Introduction

Food waste is a major challenge in modern society, caused either by spoilage or by the common belief that food past its “best by” date should be thrown away, even if it’s still safe to eat (Neff et al. 2015; Petchwattana et al. 2021). The spoilage of food can be caused by several factors at different stages from farm to fork; ranging from physical damage, natural enzyme activity, damage from the presence of microbial contaminants, and changes caused by chemicals or microbial metabolites (Rawat 2015; Shwaiki et al. 2022). Microbial food spoilage is a well-researched aspect of food production, as various microorganisms can cause spoilage under favourable conditions (Gram et al. 2002). Today, preserving food has advanced and diversified in terms of the methods used, such as applying low temperatures, heat, vacuum packaging, reducing the water content, acidification and the most well-known, chemical preservation (Shwaiki et al. 2022). In recent years, there has been an increasing concern among consumers regarding the safety of synthetic chemical preservatives used in food products. This growing awareness has sparked a search for natural alternatives, particularly those that offer antimicrobial properties. Among the natural solutions being explored are the components derived from medicinal plants, such as EOs, which are known for their potent antibacterial and antioxidant characteristics (Mancuso et al. 2019; Jahadi et al. 2021). These natural compounds are increasingly seen as safer options in food preservation (Nakhaee Moghadam et al. 2020). However, despite their promising properties, most EOs face significant limitations when it comes to stability. They are highly sensitive to environmental factors such as oxygen, light, and temperature, making them prone to degradation. In addition, some EOs exhibit strong flavour and aroma, which can alter the sensory properties of food, posing challenges to their direct use in food applications (Dimopoulos et al. 2021). To address these challenges, encapsulation has emerged as one of the most effective techniques for preserving the beneficial characteristics of EOs.

Through microencapsulation, the compound to be preserved is almost completely isolated from external factors. This microencapsulation masks undesirable odours, prevents the evaporation of volatile aromatics, and protects photosensitive components from light and oxygen, thereby drastically reducing oxidative degradation (Dimopoulos et al. 2021; Gottardo et al. 2022). Moreover, it provides greater control over the release mechanism of the essential oil, enhancing the stability of the encapsulated compounds and extending the shelf life of the final product (Salari et al. 2015; Liu et al. 2017; Kavetsou et al. 2019). There are a variety of encapsulation techniques available for EOs, including spray drying, coacervation, liposome entrapment, inclusion complexation, crystallization, nanoencapsulation, freeze-drying, emulsions and yeast encapsulation (Sultanbawa 2011; Nakhaee Moghadam et al. 2020). Among these methods, yeast encapsulation is gaining attention as an innovative and promising approach. Its appeal as a carrier lies in its low cost, ubiquity, and the unique structure of yeast cells, which offers distinct functional and nutritional benefits (Akinyemi et al. 2022; Shruthi et al. 2022). The thick (100–200 nm), phospholipid-based cell membrane structurally resembles a liposome, enabling the effective encapsulation of both hydrophobic and hydrophilic compounds. This versatility makes yeast suitable for a wide range of food ingredients and pharmaceutical actives (Paramera et al. 2011; Nakhaee Moghadam et al. 2021). Furthermore, the yeast cell wall, which consists of a beta-glucan network, chitin, and a mannoprotein layer, enhances its functionality compared to other encapsulation carriers (Salari et al. 2015). This porous structure facilitates the efficient diffusion and transport of bioactive molecules (Dimopoulos et al. 2021). The species *Saccharomyces cerevisiae* represents one of the most commercially significant yeast species, widely utilized in the food industry for processes such as baking, brewing, ethanol production, and as a source of biomass (Dimopoulos et al. 2021). Its neutral, creamy-white colour and bland taste make it an organoleptically unobtrusive ingredient in food products (Dadkhodazade et al. 2018). Importantly, most strains of *S. cerevisiae* are classified as Generally Recognized as Safe (GRAS), further solidifying its suitability for use in food preservation and related applications (Kavetsou et al. 2019; Coradello and Tirelli 2021; Nakhaee Moghadam et al. 2021). To incorporate secondary products within *S. cerevisiae* cells, their disruption is a necessary initial step (Dimopoulos et al. 2021). Autolysis is a standard method used to increase cellular permeability or to fully rupture the cells, thereby releasing intracellular components. In the food industry, autolysis is typically carried out at around 50 °C and pH 5.5. During this process, the cell wall is primarily altered in permeability but generally retains its structural shape, research shows the plasma membrane, not the cell wall, sustains the major lytic damage (Dimopoulos et al. 2021).

Recent research has focused on encapsulating EOs within yeast cells to develop natural, bio-based carriers with functional properties such as larvicidal and insecticidal activity. For instance, (Kavetsou et al. 2019) demonstrated that *Mentha pulegium* EO encapsulated in yeast cells, with an encapsulation efficiency of 29—36%, exhibited a stronger pesticidal effect against *Myzus persicae* than its free counterpart. Similarly, this carrier system has shown promise in food applications:(Nakhaee Moghadam et al. 2020) found that yeast-encapsulated *Zataria multiflora* EO acted as an effective antimicrobial against *E. coli* and *L. monocytogenes* in chicken soup while also masking the oil's potent odor. Further supporting its utility in food preservation, (Gottardo et al. 2022) reported that a 50:50 blend of microencapsulated oregano and cinnamon EOs significantly reduced *Listeria monocytogenes* in Italian salami without adversely affecting texture. (Liu et al. 2021) showed that yeast-encapsulated Mānuka EO achieved a 4-log CFU/mL reduction against *Bacillus cereus* under high organic load, whereas non-encapsulated oil yielded less than a 1-log reduction. While EO encapsulation via techniques like emulsification, coacervation, spray drying, and complexation is well-documented, the specific use of yeast cells as carriers remains relatively underexplored, particularly for food systems. Moreover, our review of the literature indicates a clear research gap: the encapsulation of cinnamon, thyme, and oregano essential oils within yeast cells specifically for preserving meat products like ham remains unexplored. This study therefore introduces a novel approach to address this gap, aiming to leverage yeast encapsulation for improved, natural food preservation. To address this gap, the present study tested the hypothesis that encapsulating essential oils within *S. cerevisiae* cells improves their stability and antimicrobial performance by protecting the bioactive compounds from environmental degradation, and that yeast-encapsulated EOs provide greater preservation of meat than equivalent concentrations of free EOs. The specific objectives were to screen EOs for antimicrobial activity and select the most active; to optimise the encapsulation conditions; to compare encapsulation in autolysed and non-autolysed yeast cells; to determine the minimum inhibitory concentration and antimicrobial activity of the encapsulated EOs; and to evaluate their effectiveness in extending the shelf-life of ham under controlled storage.

## Materials and methods

### Plant materials and reagents

Essential oils of cinnamon leaf (*Cinnamomum zeylanicum*), oregano (*Origanum vulgare*), rosemary (*Rosmarinus officinalis*), and thyme (*Thymus vulgaris*) were procured from a commercial supplier in the UK. All oils were stored in airtight, light-proof containers at 4 °C to prevent degradation prior to analysis. Pure dry yeast (*S. cerevisiae*), peptone water, Eugenol, (99%), Carvacrol (99%), Camphor (96%), Dimethyl Sulfoxide (DMSO) Mueller Hinton Agar and Broth (MHA, MHB), Potato Dextrose Agar (PDA, Oxoid), Tryptic Soy Agar (TSA, Oxoid) and Tryptone Bile X-Glucuronide (TBX, Oxoid) agar were obtained for microbiological and encapsulation work. All chemicals and solvents, including n-hexane and absolute ethanol, were of analytical grade.

### Selection of essential oils based on antimicrobial screening

A preliminary confirmatory screening was conducted to evaluate the antimicrobial potential of nine candidate essential oils: angelica root, clove bud, cinnamon leaf, ginger, lemongrass, oregano, rosemary, sage, and thyme. Antimicrobial activity was assessed using the agar well diffusion method against two bacterial strains *Escherichia coli* NCTC 9001 and *Enterococcus faecium* ATCC 8459 and two fungal species *Aspergillus niger* and *Penicillium aerogenes*.

Bacterial cultures were propagated from single freeze-dried discs in Tryptic Soy Broth (TSB) at 37 °C for 18–24 h. Purity was confirmed by triple subculturing on selective media: TBX agar for *E. coli* and TSA for *E. faecium*. Fungal spores were collected from established cultures on PDA incubated at 25 °C for 3–5 days. Microbial suspensions were adjusted to a 0.5 McFarland standard (1.5 × 10⁸ CFU/mL) using a Wickerham card for turbidimetric standardization. For the assay, 100 µL of standardized suspension was spread onto MHA (bacteria) or PDA (fungi) to form confluent lawns. Wells (8 mm diameter) were aseptically prepared and filled with 50 µL of each essential oil. Plates were pre-incubated at 10–15 °C for 3 h to allow oil diffusion, followed by incubation at 37 ± 2 °C for 24 h (bacteria) or 25 °C for 128 h (fungi). All tests were performed in triplicate. Inhibition zones were measured using a calibrated digital calliper. Based on quantitative efficacy, three oils demonstrating the strongest antimicrobial activity and one with negligible activity were selected for subsequent phases.

### Density measurement and chemical profiling of essential oils

The density of each essential oil was measured in triplicate at 20 °C using an Anton Paar DMA 4100 M densitometer. The mass of oil contained in a 50 µL volume was subsequently calculated from the measured density using the formular below.

$$m \left(mg\right)=d \left(g/mL\right)\times V(\mu L)$$

where m = Mass, d = density, V = volume.

Chemical composition was analysed by gas chromatography–mass spectrometry (GC–MS) using a Shimadzu GC/MS-QP 2010 S equipped with a SUPELCOWAX™ 10 capillary column (30 m × 0.25 mm, 0.25 µm). Helium carrier gas flow was maintained at 1 mL/min. The oven temperature programmed to increase from 40 °C to 250 °C at a rate of 10 °C/min and held at 250 °C for 12 min. The injector and detector temperatures were consistently maintained at 250 °C. Volatile compounds in each EO were adsorbed using a solid-phase microextraction (SPME) fibre (PDMS/DVB Fibre P; polydimethylsiloxane/divinylbenzene coating) and subsequently added into the GC–MS injection port. Samples were introduced into the GC/MS system through the headspace injection set at split mode (100:1 ratio), with a 1.0 µL aliquot of each EO placed in a 20 mL headspace vial. The sealed vial was equilibrated at 40 °C for 30 min in a heating block prior to analysis. Mass spectra were acquired across a mass-to-charge (m/z) range of 33–500, with an ionisation energy of 70 eV. Compound identification was performed by cross-referencing retention indices with the National Institute of Standards and Technology (NIST) 2011 mass spectral library, supplemented by the Babushok and Mass Finder databases (Babushok et al. 2011).

### Encapsulation of essential oils in saccharomyces cerevisiae

Yeast suspensions were prepared by dissolving pure dry yeast in deionized water at 10% (w/w) concentration. The mixture was gently stirred for 20 min at room temperature to allow the yeast cells to rehydrate. Following rehydration, the suspension was split into two batches of triplicates, batch A for none treated cells (control) and batch B for autolysis treatment. The batch A yeast cells suspensions were centrifuged at 3220 g for 10 min. The supernatant was aspirated and discarded, while the cell pelleted residues were collected and lyophilized using a freeze dryer (Benchtop, Spain) at −55 °C for 48 h (Dimopoulos et al. 2021). Autolysis was conducted on batch B samples in a sealed 1-L borosilicate glass reactor to create empty, hollow intracellular spaces that can act as reservoirs to trap and protect essential oils. Before starting, the pH of the yeast cell suspension was standardized to 5.5 through titration with 0.1 M NaOH and 0.1 M acetic acid. The suspension was subsequently subjected to mechanical autolysis under controlled conditions: incubation for 24 h in a temperature-regulated orbital shaker bath (52 °C ± 0.5 °C) with continuous agitation at 170 rpm. Post-autolysis, the lysate was centrifuged (3220 g, 20 min) to separate insoluble cellular debris from the hydrolysed fraction. The supernatant was aspirated and discarded, while the cell pellet residues were collected and lyophilized.

The methodology was adapted from Bishop et al (1998) with slight modifications to optimise compound retention. Lyophilised yeast cell material from both autolyzed and non-autolyzed treatments was reconstituted in a homogenised mixture comprising (w/w): 14.5% cell biomass, 14.5% EO, 63.8% sterile deionised water, and 7.2% ethanol. The inclusion of ethanol was for two reasons: first, to reduce the interfacial tension between the EO and the aqueous phase, promoting emulsification; and second, to mildly disrupt the yeast cell wall structure, enhancing permeability without compromising its integrity. Following homogenisation, this formulation was incubated in 1-L borosilicate glass reactors under controlled conditions on a temperature-regulated orbital shaker bath (45 °C ± 0.5 °C) with constant agitation at 170 rpm for 15 min. Post-incubation, aliquots were immediately centrifuged (3220 g, 20 min) to separate encapsulated fractions. The supernatant, containing unbound EO residues, was decanted and discarded. Yeast cells encapsulated with EO were subjected to three gentle successive wash cycles with deionised water (1:10 w/v) to eliminate residual weakly bound or non-encapsulated oil. Finally, the washed capsules were lyophilised using the protocol discussed above.

### Morphological and spectrophotometric confirmation of encapsulation

The morphological analysis of the empty and EO microcapsule was evaluated in a Scanning Electron Microscope (SEM Vega LM3, Tescan, Czech Republic). The samples were spread on a stub with a double carbon tape and coated (Q150 R ES, Quorum) with a thin 10 μm gold layer. Images were taken at 10 kV.

For analytical characterization of encapsulated essential oils, a sequential extraction and spectroscopic protocol was implemented. Freeze-dried yeast-encapsulated EO (20 g) underwent ethanol-mediated extraction (70% ethanol) with ultrasonic assistance (40 kHz). Post-centrifugation (3220 × g, 10 min), the hydrophobic EO phase was isolated from the aqueous-ethanol interface. Comparative UV–Vis spectrophotometric analysis (200–800 nm) between encapsulated and free EO fractions was performed to assess encapsulation-related spectral changes.

### Quantification of total, surface bound and encapsulated essential oil.

The total EO content within encapsulated yeast cells were determined by quantifying both surface-adhered and intracellularly encapsulated EO. A hybrid protocol, adapted from Bishop et al (1998) and Dimopoulos et al (2021), was employed to quantify total EO content (encapsulated and surface-bound). Lyophilised yeast encapsulates (50.0 mg ± 0.001 g) were homogenised in 500 μL deionised water using a vortex mixer (5 min, 2500 rpm). Absolute ethanol (4.5 mL) was added to solubilise hydrophobic EO constituents, followed by secondary vertexing (5 min) and centrifugation (4000 rpm, 10 min). The supernatant was filtered (0.2 μm PTFE syringe filter), diluted 1:10 (v/v) with absolute ethanol, and quantified using GC–MS (1 μL injection volume). The total EO concentration was calculated using an internal standard calibration curve with the final values adjusted by a 10 × dilution factor and the percentage of the predominant bioactive compound in each EO, expressed as mg EO/100 mg yeast cell.

Surface-bound EOs were recovered by suspending lyophilized encapsulates (50.0 mg ± 0.001 g) in 5 mL n-hexane, a non-polar solvent. The suspension was vortexed (30 s, 2500 rpm) to dislodge surface-bound oils and centrifuged (4000 rpm, 10 min). The hexane phase was filtered (0.2 μm PTFE syringe filter) and the resulting solution injected (1 μL) into the GC–MS system. The surface-bound EO concentration was calculated using an internal standard calibration curve with the final values and the percentage of the predominant bioactive compound in each EO, expressed as mg EO/100 mg yeast cell.

The EO encapsulation efficiency (EF) of the yeast cells were determined using the following formular

$$\mathrm{E}\mathrm{F} (\mathrm{\%}) = \frac{Me}{Mc}=\mathrm{E}\mathrm{F} (\mathrm{\%}) = \frac{Me}{Mc}=\frac{Mt-Ms}{Mc}\times 100$$

where;

- EF: encapsulation efficiency
- Me: mass of encapsulated essential oil
- Mc: mass of yeast cell (100 mg)
- Mt: mass of total essential oil
- Ms: mass of surface-bound essential oil

### Calibration and quantification of bioactive compounds

Calibration standards of carvacrol (oregano), thymol (thyme), camphor (rosemary), and eugenol (cinnamon) were prepared in absolute ethanol (0–350 mg/mL). GC–MS analysis was performed under the conditions described in above, with split ratio 10:1. Calibration curves (peak area vs. concentration) presented as Fig S1 were constructed for quantitative analysis. The linearity of the standard curves was assessed to confirm the reliability and reproducibility of the analytical method.

### In vitro* antimicrobial efficiency of encapsulated essential oil*

For the selected essential oils, antimicrobial activity was further quantified using a standardized broth microdilution method in 96-well plates. Bacterial suspensions were prepared to a density of approximately 10^5^ CFU/mL in Mueller–Hinton broth. In each well, 100 µL of bacterial suspension was combined with 100 µL of a serial dilution of the essential oil, previously solubilized in dimethyl sulfoxide (DMSO) and diluted in broth. Final DMSO concentrations did not exceed 1% (v/v), which served as the negative growth control. Wells containing broth with 70% ethanol served as a positive sterility control, and wells with bacteria and broth alone served as a positive growth control. Plates were incubated at 37 ± 1 °C for 24 h. Following incubation, bacterial growth was assessed by measuring the optical density at 600 nm (OD₆₀₀). The minimum inhibitory concentration (MIC) was determined as the lowest concentration of essential oil that completely inhibited visible growth. All tests were performed in triplicate.

### In Situ* antibacterial activity in ham model*

The antibacterial efficacy of free and encapsulated cinnamon, oregano, and thyme EOs was evaluated in a uncooked, uncured ham model against *Escherichia coli* over a 6-day storage period. *E. coli* was selected for the *in-situ* study because it is a key indicator of the hygienic quality of meat products and a common agent of foodborne contamination. Preservative-free ham was aseptically cut into 4 × 4 cm portions using sterile surgical blades under laminar airflow. The ham used had a water activity (aw) of 0.88 and pH range between 5.1—5.5. Each portion was surface-inoculated with 100 µL of an *E. coli* suspension (0.5 McFarland standard). Subsequently, portions were treated with encapsulated EOs at doses equivalent to their predetermined minimum inhibitory concentrations: oregano (8.54 mg, equal to 3.12 µL free EO), thyme (23.06 mg, equal to 6.25 µL free EO), and cinnamon (8.41 mg, equal to 3.12 µL free EO). Parallel treatments with corresponding free EOs served as positive controls. Untreated, inoculated samples were used as negative controls, following the methodology of Hadian et al. (2017). Treated samples were stored under both vacuum-sealed and non-vacuum conditions. Bacterial viability was assessed daily. At each time point, 10 g of ham was homogenized in 90 mL of peptone water, serially diluted, and plated on TBX chromogenic agar. After incubation at 37 °C for 24 h, colonies were enumerated. All experiments were performed in triplicate.

### Physicochemical analysis

A series of physicochemical analyses was conducted to evaluate the quality attributes of raw ham throughout the storage period. The assessed parameters included colour, water activity (aw), and texture. Untreated raw ham was analysed on Day 1 as the control to establish baseline values for comparison. At the end of the storage period, the same analyses were performed on ham samples treated with EO. Colour was measured using a Hunter colorimeter (Konica Minolta CR-400, Osaka, Japan), calibrated with a CIE D65 standard illuminant to ensure accurate and consistent measurements. The colour parameters recorded were L* (0 = black, 100 = white), a* (− green to + red), and b* (− blue to + yellow). Water activity (aw) was determined using an AQUALAB 4TE water activity meter (USA) at 24.5 ± 0.5 °C. This parameter was measured to assess moisture availability in the ham samples, which is an important factor influencing microbial growth, enzymatic activity, and shelf-life stability. Texture was evaluated using a TA. XTplus texture analyser (Stable Micro Systems, England) equipped with a knife and shear probe. Ham samples (8 × 8 cm) were subjected to a shear force test, and the maximum cutting force required to penetrate each sample was recorded as an indicator of firmness and structural integrity during storage. All measurements were performed in triplicate.

### Statistical analysis

All statistical analyses were conducted using R statistical software (version 4.5.0) with packages including *lme4, survival, emmeans*, and *multcomp*. Significance was established at α = 0.05 for all analyses, with p-values reported to three decimal places. Effect sizes were calculated where appropriate (Cohen's d for t-tests, η^2^ for ANOVAs, C-index for survival models) to provide quantitative measures of practical significance alongside statistical significance. The experimental design incorporated factorial arrangements with multiple independent variables to assess their effects on essential oil encapsulation and antimicrobial efficacy. For encapsulation efficiency studies, a 4 × 2 factorial design was employed, comprising four essential oils (cinnamon, oregano, thyme, rosemary) and two treatment conditions (treated/untreated yeast cells), with triplicate measurements (n = 3) per combination. Antimicrobial efficacy investigations utilized a 3 × 2 × 2 factorial design encompassing three essential oils (cinnamon, oregano, thyme), two formulations (free vs. encapsulated), and two packaging conditions (vacuum-sealed vs. non-sealed), with temporal measurements every 24 h for 6 days, each with six biological replicates (n = 6).

All continuous variables (encapsulation efficiency percentages, inhibition zone diameters, CFU counts) were initially subjected to descriptive statistical analysis, including calculation of means and standard deviations. CFU count data underwent logarithmic transformation (log₁₀[CFU + 1]) to normalize distributions. All transformations were validated using Shapiro–Wilk tests for normality and Levene's tests for homogeneity of variances.

Differences in encapsulation parameters (total retention, surface binding, internal encapsulation) between treated and untreated conditions for each essential oil were evaluated using paired Student's t-tests. For comparisons across multiple essential oils, one-way analysis of variance (ANOVA) was performed, followed by Tukey's Honestly Significant Difference (HSD) post-hoc tests for pairwise comparisons when significant main effects were detected (p < 0.05). Two-way ANOVA was employed to examine interactions between essential oil type and treatment condition, with significant interactions further investigated using simple main effects analysis.

Minimum inhibitory concentration determinations were analysed using non-parametric approaches due to the ordinal nature of dilution series data. The Mann–Whitney U test was applied to compare MIC values between free and encapsulated formulations for each essential oil. For zone of inhibition measurements at different concentrations, two-way repeated measures ANOVA was conducted with formulation and concentration as independent variables. Multiple comparisons were adjusted using the Holm-Bonferroni method to control family-wise error rates.

Bacterial survival data from storage studies were analysed using mixed-effects linear models with log₁₀-transformed CFU counts as the response variable. Fixed effects included formulation, packaging condition, storage temperature, time, and their interactions, while random effects accounted for replicate variability. Model selection was based on Akaike Information Criterion (AIC) comparison and likelihood ratio tests. Time-to-event analysis was conducted using Kaplan–Meier survival estimates with log-rank (Mantel-Cox) tests to compare survival distributions between treatment groups.

## Result

### Antimicrobial activity of selected essential oil

**Fig. 1:**
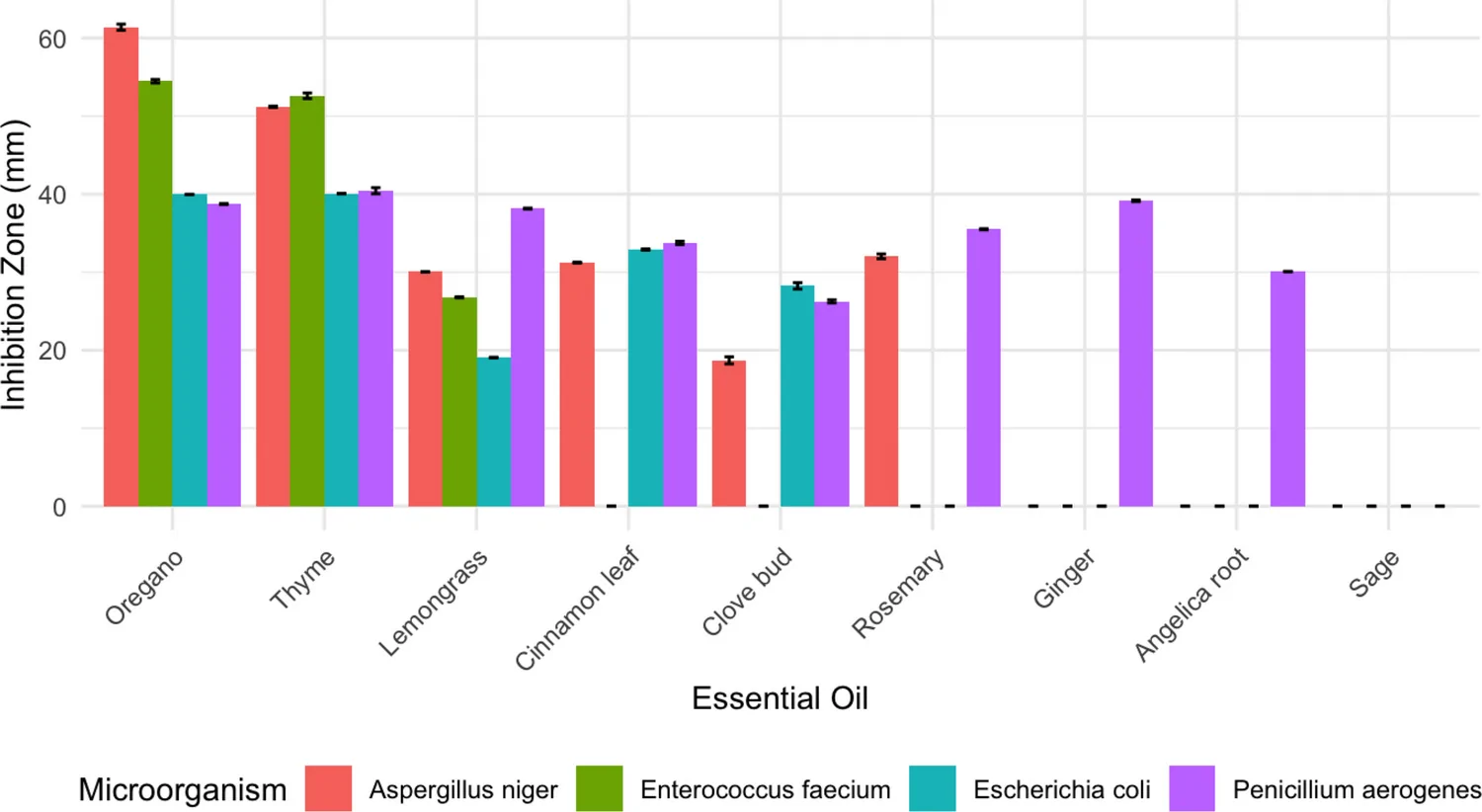
Antibacterial and Antifungal Activity of Essential Oils Measured by Agar Well Diffusion Assay. Zones of inhibition (mm) for nine essential oils against Gram-negative (*Escherichia coli*) and Gram-positive (*Enterococcus faecium*) bacteria, and fungal species (*Aspergillus* *niger, Penicillium aerogenes*). Values represent mean inhibition zone diameter (mm) from triplicate experiment

The antimicrobial efficacy of nine essential oils angelica root, clove bud, cinnamon leaf, rosemary, sage, lemongrass, oregano, ginger, and thyme was quantitatively assessed against *E. coli*, *E. faecium*, *A.* *niger*, and *P. aerogenes* using the agar well diffusion assay. The results, summarised in Fig. 1, demonstrate variations in inhibitory activity among the selected essential oils across the tested microorganisms. Oregano and thyme essential oils exhibited broad-spectrum antimicrobial activity, with oregano producing the largest zones of inhibition against *E. faecium* (54.47 mm), *A.* *niger* (61.6 mm), and *P. aerogenes* (38.55 mm), while also showing strong activity against *E. coli* (40 mm). Thyme essential oil demonstrated similarly high efficacy against *E. faecium* (52.46 mm) and *A.* *niger* (51.24 mm). Cinnamon leaf essential oil showed pronounced activity against fungal strains, particularly *A.* *niger* (31.18 mm) and *P. aerogenes* (33.53 mm). Interestingly, *E. faecium* exhibited resistance to most essential oils, with significant inhibition observed only for oregano and thyme. Sage essential oil displayed no detectable antimicrobial activity against any of the tested strains, while angelica root and ginger showed limited efficacy, with activity only against *P. aerogenes*. Essential oils of cinnamon leaf, oregano, and thyme which demonstrated the most significant inhibitory activity were selected for further investigation. For comparative purposes, rosemary essential oil which exhibited negligible activity was also included in subsequent phases of the research.

### Density and major chemical constituents of selected essential oils

The measured densities and corresponding calculated masses for the four essential oils, critical for standardizing application quantities in subsequent antimicrobial assays, are presented in Table 1. The density (d) of each oil was determined at 20 °C using an oscillating U-tube density meter, yielding values of 1045.6 ± 0.10 kg m⁻^3^ for cinnamon leaf oil, 966.8 ± 0.14 kg m⁻^3^ for oregano oil, 812.4 kg m⁻^3^ for thyme oil, and 910.23 ± 0.06 kg m⁻^3^ for rosemary oil. Using these densities, the mass (M) of a fixed application volume (V) of 50 µl was calculated for each oil, resulting in masses of 52.28 mg, 48.34 ± 0.01 mg, 40.62 mg, and 45.51 mg for cinnamon leaf, oregano, thyme, and rosemary oils, respectively. Consequently, all subsequent quantitative analyses expressed essential oil quantities in mass units (mg) rather than volume to ensure consistency and accuracy in reporting.

**Table 1:** Density and calculated mass per volume for essential oils used in antimicrobial testing

Essential oil	d (20/20) °C	V (µl)	M (mg)
Cinnamon leaf	1045.6 ± 0.10	50	52.28
Oregano	966.8 ± 0.14	50	48.34 ± 0.01
Thyme	812.4	50	40.62
Rosemary	910.23 ± 0.06	50	45.51

Chromatographic analysis successfully characterized the bioactive constituents of each essential oil. The ten most abundant compounds for each oil are presented in Table 2, while the complete, detailed compositional profiles are available in Data S1. Cinnamon oil (CIN) was characterised by a high abundance of eugenol, which exhibited a peak area of 31,435,494 and a retention index (RI) of 1333 at 12.71 min, followed by caryophyllene (RI 1424.57) and linalool (RI 1085). Oregano oil (ORE) was overwhelmingly dominated by carvacrol, displaying an exceptionally large peak area of 54,285,676 with an RI of 1280.72 at 11.978 min. Significant contributions were also noted from *m*-cymene (RI 1013) and γ-terpinene (RI 1051.34). In rosemary oil (ROS), the major constituents were camphor (peak area 33,897,909, RI 1125), eucalyptol (RI 1022.54), and α-pinene (RI 933). The profile for thyme oil (THY) was heavily defined by thymol, which had a peak area of 58,913,132 and an RI of 1271.6 at 11.844 min, with terpinolene (RI 1081) as a secondary major compound. The calculated retention indices for these primary bioactive compounds showed strong concordance with reference values from the NIST MS Library 2011, confirming their identity. Notably, the compositional profiles for rosemary and thyme included several alkane derivatives among their top ten constituents, whereas cinnamon and oregano profiles were comprised entirely of characteristic phenolic and terpenoid compounds. Expressed as relative peak-area percentages of the total volatile profile, the predominant constituent accounted for 50.3% (eugenol) in cinnamon, 61.9% (carvacrol) in oregano, 30.8% (thymol) in thyme, and 15.6% (camphor) in rosemary oil, the lower phenolic percentages in thyme and rosemary reflecting the large share of co-eluting monoterpene and alkane constituents in those oils; the full relative compositions (%) are provided in Data S1 and, for the major constituents, in Table 2. The chemical structures of the principal constituents of each oil are shown in Table 2, providing a structural basis for the differences in bioactivity and encapsulation behaviour discussed below.

**Table 2:**
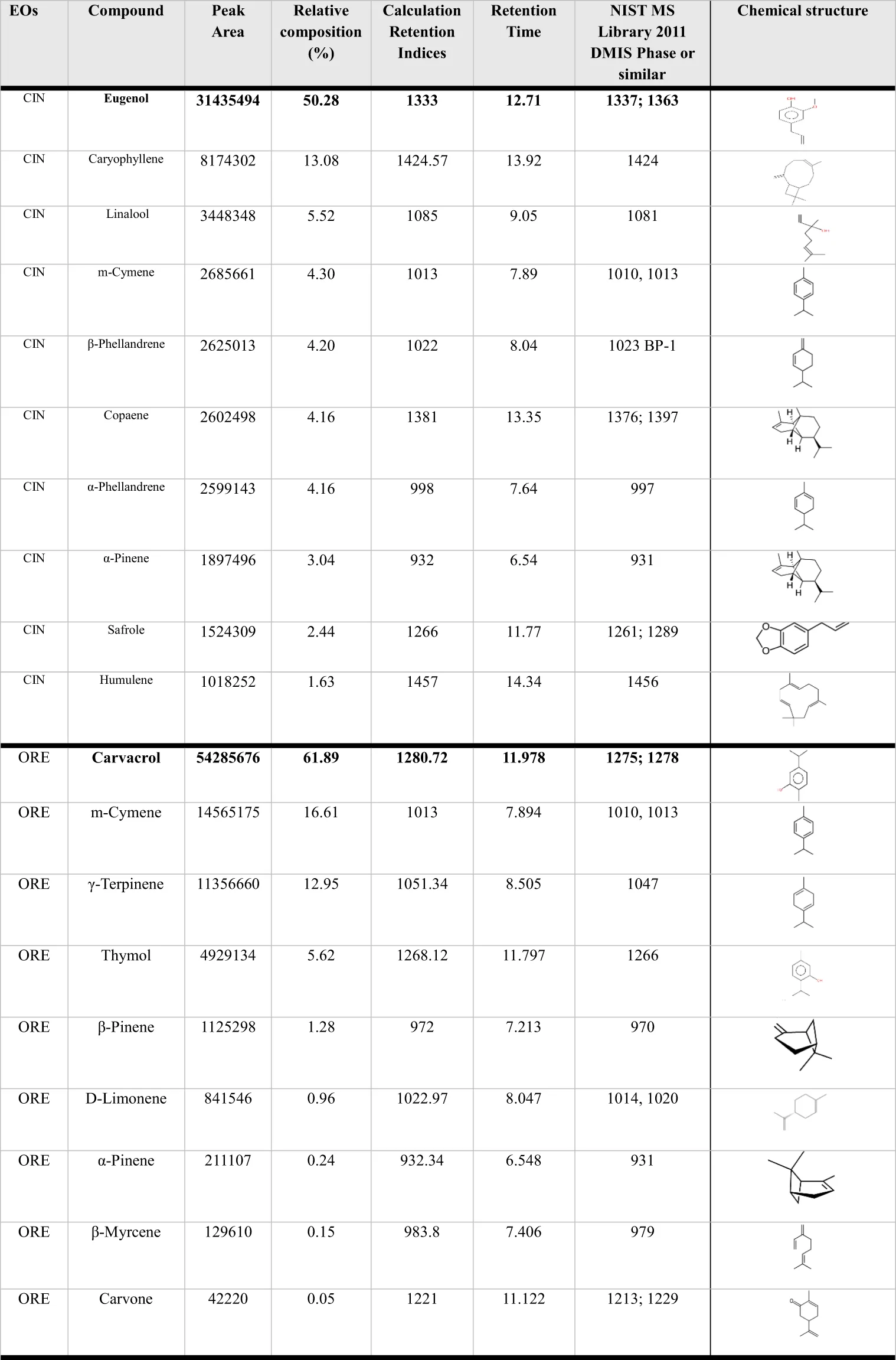

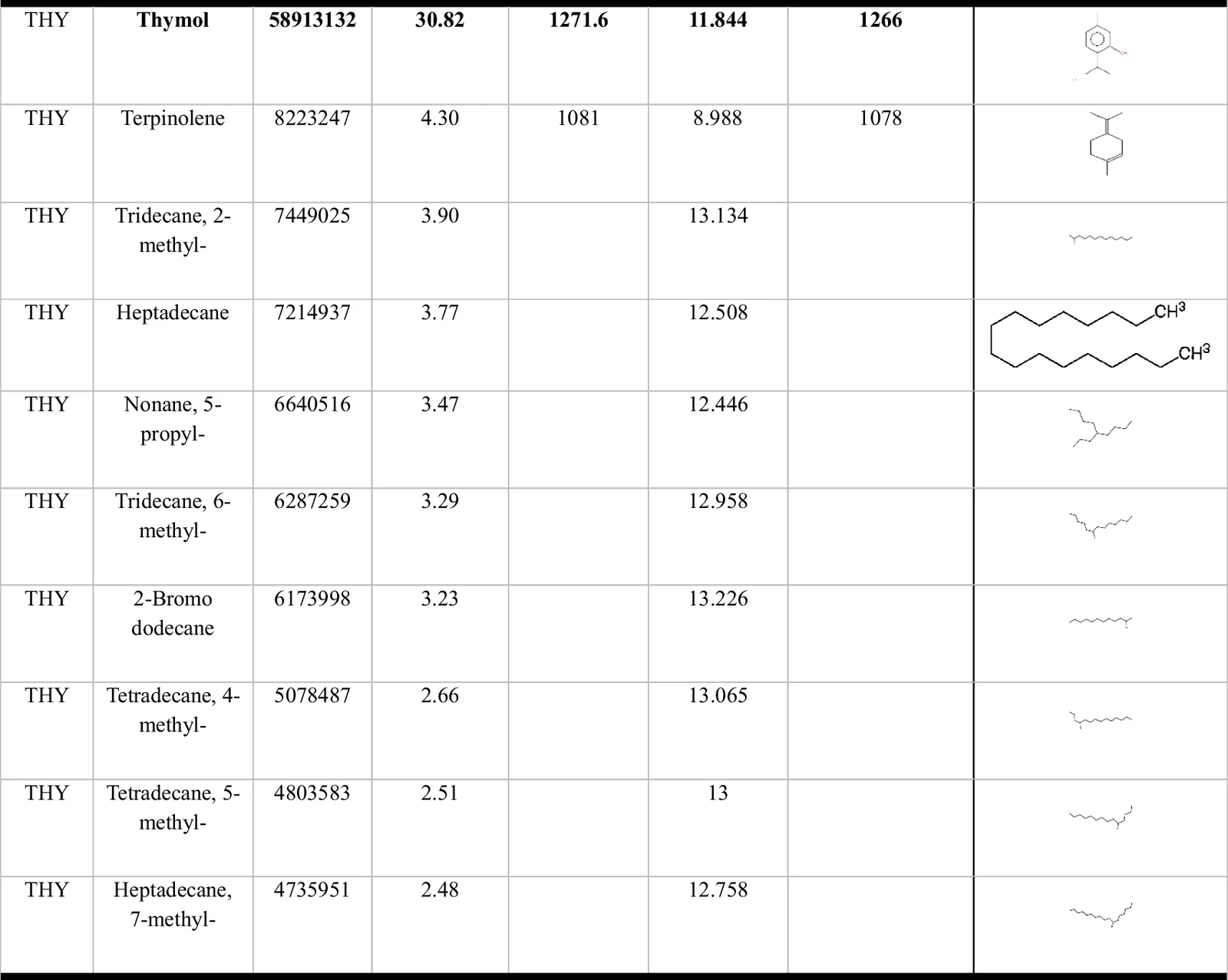

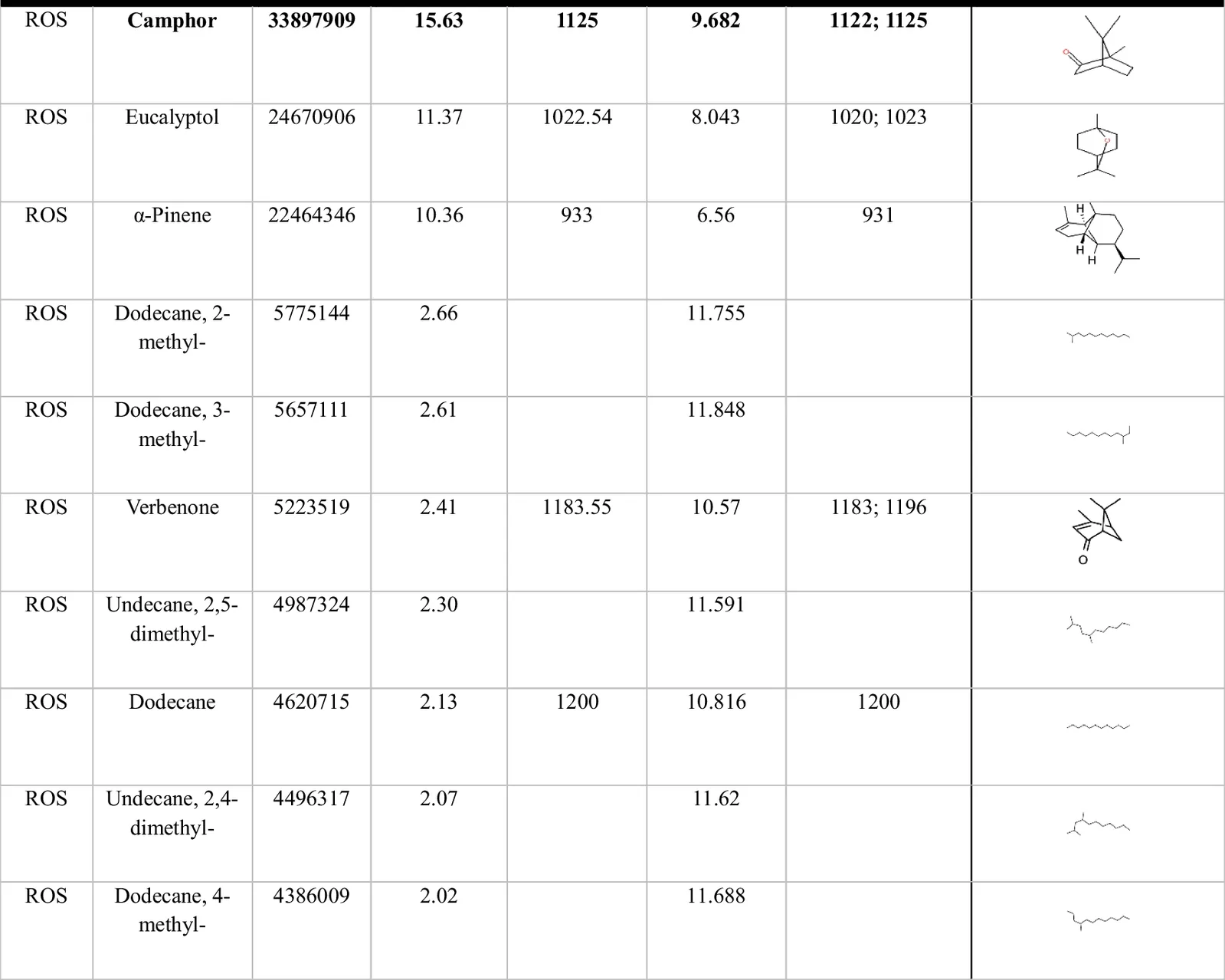
Major chemical constituents of cinnamon (CIN), oregano (ORE), rosemary (ROS), and thyme (THY) essential oils identified by gas chromatography-mass spectrometry (GC–MS) analysis. The table presents peak areas, calculated and reference retention indices (Kovats indices), retention times, and matches with the NIST 2011 MS library database. Compounds are listed in descending order of peak area for each oil. Representative GC–MS chromatograms of the four essential oils selected for encapsulation are shown in Fig. S2

### Essential oil encapsulation in yeast cell

**Fig. 2:**
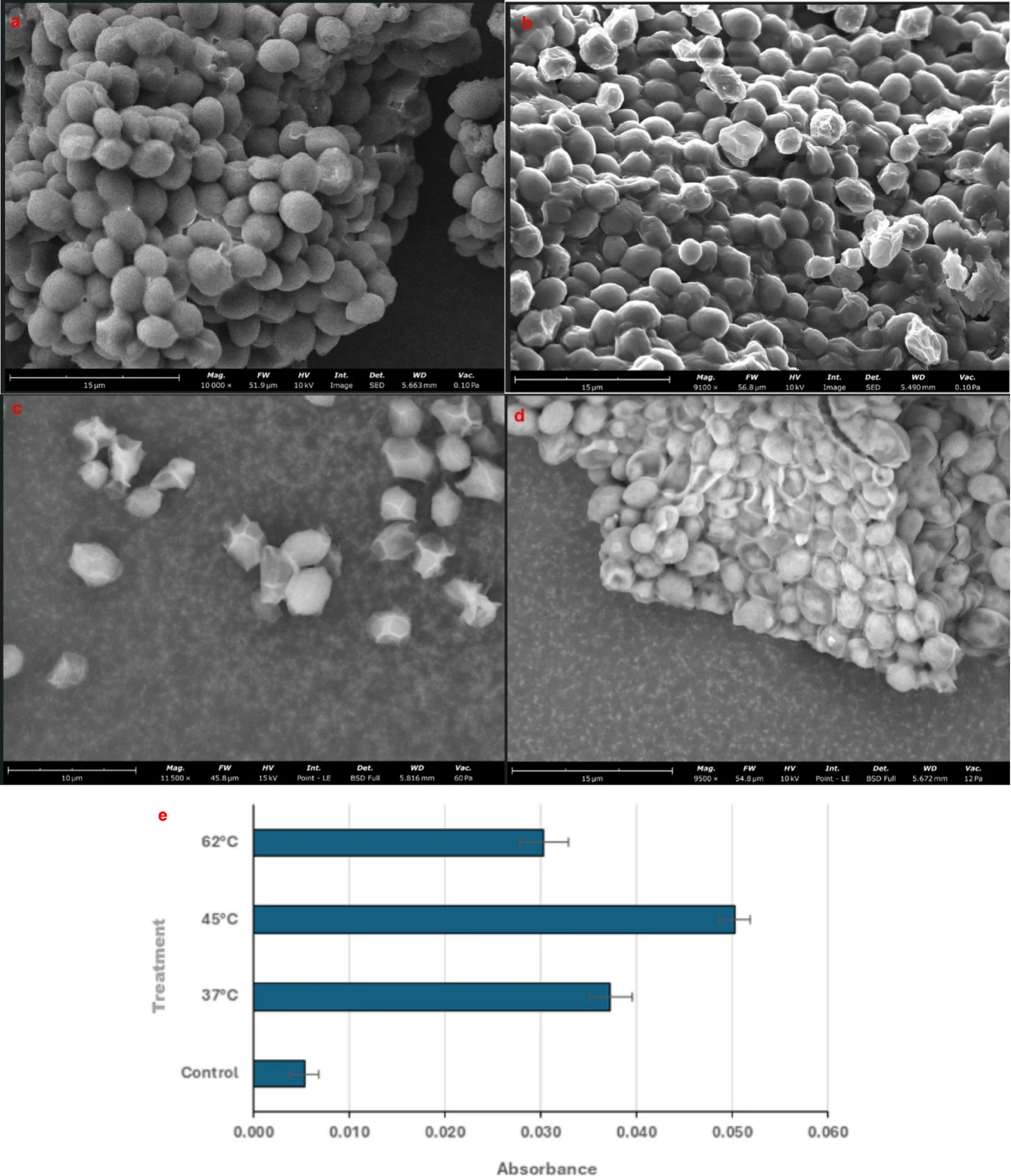
Scanning electron micrographs of yeast cells at different autolysis temperatures. Images show (**A**) untreated yeast cells, and yeast cells after autolysis at (**B**) 37 °C, (**C**) 45 °C, and (**D**) 62 °C. All micrographs were acquired at magnifications ranging from 9,000 × to 11,500 ×. Scale bars are included in each panel. (**E**) Absorbance of the essential oil recovered from yeast cells loaded at 37 °C, 45 °C, and 62 °C, together with the untreated control, used as an indicator of oil-loading efficiency; bars represent the mean and error bars the standard deviation of triplicate measurements

The optimal temperature for essential oil encapsulation in *S. cerevisiae* was evaluated based on three factors; cellular integrity, post-encapsulation viability, and oil retention capacity after encapsulation at 37 °C, 45 °C, and 62 °C. Cell viability assessment via plate counting confirmed preserved reproductive capacity in encapsulated yeast samples treated at 37 °C and 45 °C, evidenced by robust colony formation after 24 h incubation (Fig S3). No viable colonies were detected following 62 °C treatment. Scanning electron microscopy (SEM) was employed to analyse morphological and structural changes in yeast cells under the different encapsulation conditions. Representative micrographs captured at 10,000 × magnification (Fig. 2) revealed distinct temperature-dependent responses. Control cells (untreated) exhibited characteristic ovoid morphology with smooth, intact surfaces and typical cluster arrangements (Fig. 2a). Cells encapsulated at 37 °C maintained structural integrity with minimal deformation, indicating effective essential oil absorption without significant wall damage (Fig. 2b). At 45 °C, moderate thermal stress was evident through surface wrinkling and membrane folding, though overall cellular integrity remained preserved (Fig. 2c). In contrast, 62 °C treatment caused severe structural deterioration characterized by collapse, fragmentation, and extensive surface crevices, indicating irreversible thermal degradation (Fig. 2d). It is important to state that non-viable (dead) yeast cells can also serve as empty carrier shells, so cell viability is not, in itself, a requirement for encapsulation. In this study, viability test and microscopic characterisation were used to confirm that the cell wall and membrane remained intact; at 62 °C the cells lost viability and collapsed structurally, which is probably why there was reduction in volume of retained the oil. The optimal loading temperature was chosen on this combined basis of cell integrity, viability, and oil loading: loading was highest at 45 °C (Fig. 2e) with cell integrity preserved, comparable at 37 °C, and sharply reduced at 62 °C. We also considered the potential use of encapsulated yeast in the curing or fermentation of meat, where viable encapsulated cells would be industrially advantageous, contributing to the process while the encapsulated EO helps to suppress the growth of spoilage and pathogenic bacteria. For this reason, we opted for the higher viable limit of 45 °C for subsequent testing. These loading comparisons were used as a practical indicator and were not subjected to formal inferential statistical testing.

**Fig. 3:**
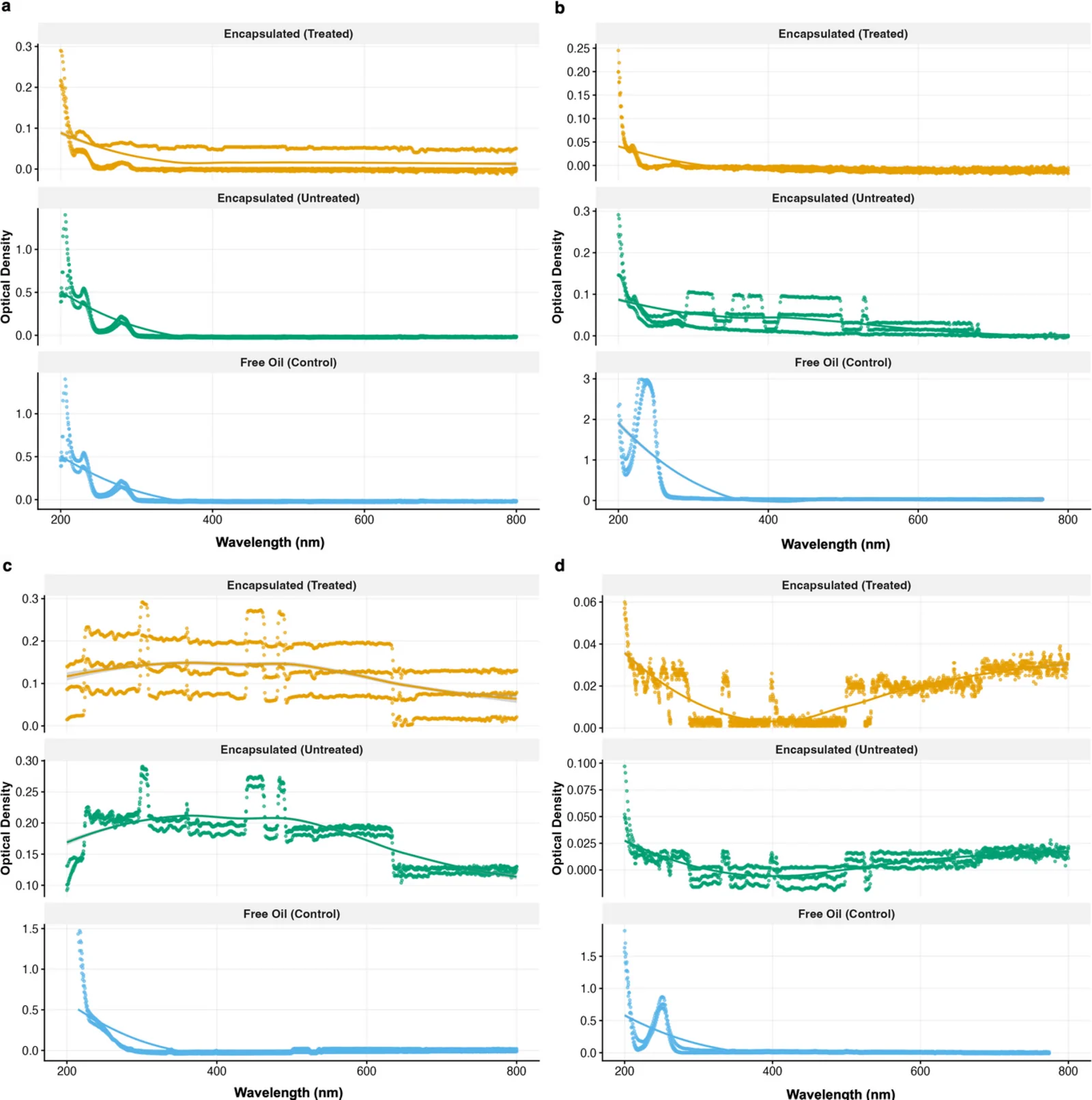
UV–Vis spectroscopic analysis of free and yeast-encapsulated essential oils. Absorbance (optical density) was recorded across the 190–800 nm range for (**A**) cinnamon, (**B**) oregano, (**C**) rosemary, and (**D**) thyme essential oils. Within each panel, spectra are shown for the essential oil encapsulated in autolysed yeast cells [Encapsulated (Treated)], the essential oil encapsulated in non-autolysed yeast cells [Encapsulated (Untreated)], and the free, non-encapsulated essential oil [Free Oil (Control)]

The UV–Vis spectroscopic analysis (Fig. 3) was carried out to evaluate and compare the encapsulation of the four selected essential oils, cinnamon (Fig. 3a), oregano (Fig. 3b), rosemary (Fig. 3c), and thyme (Fig. 3d), within *S. cerevisiae* yeast cells at 45 °C. In their free (control) form, all four oils absorbed strongly in the 200–290 nm region, a window diagnostic of the π → π* electronic transitions of aromatic rings, phenolic compounds (e.g., cinnamaldehyde, carvacrol, and thymol), and conjugated systems native to these oils; this region was therefore used to detect unencapsulated, freely dissolved oil. Free-oil absorbance was most intense for oregano, whose peak optical density (OD) exceeded 2.9, followed by thyme, rosemary, and cinnamon (peak OD ≈ 1.4–1.9).

In the autolysed (treated) yeast cells, the peak OD of cinnamon, oregano, and thyme fell below 0.3, an ~ 80–97% reduction relative to their free-oil controls, indicating that most of the oil had been sequestered within the yeast cells rather than remaining free in solution; oregano showed the largest change (peak OD from > 2.9 to < 0.25). Rosemary showed the least effective sequestration, retaining a high residual absorbance in the encapsulated cells, which was consistent with its lowest encapsulation efficiency and its predominantly non-phenolic, camphor-rich composition. A comparable, though generally smaller, reduction was observed in the non-autolysed (untreated) cells for oregano, rosemary, and thyme.

Across all four oils, successful encapsulation within the yeast matrix was indicated by the reduction in absorbance of both the treated and untreated cells relative to their free-oil controls. This was further confirmed by a sonication-based release experiment: when a separate aliquot of the encapsulated yeast cells was disrupted by sonication, the released material showed a sharp increase in optical density, restoring the absorbance to levels similar to those of the original free, unencapsulated essential oil. This recovery of the characteristic absorbance signal upon cell lysis provides direct, confirmatory evidence that the low OD of the intact encapsulated samples reflected the physical sequestration and shielding of the oils within the yeast microcarriers, rather than chemical degradation or loss of the essential oils.

### Encapsulation efficiency analysis

Encapsulation efficiency (EF) is defined as the percentage of a target compound successfully entrapped within a carrier matrix relative to the initial quantity used. In this study, EF was calculated by quantifying the total essential oil (EO) retained by yeast cells (per 100 mg), which includes both surface-bound (external) and encapsulated (internal) EO. The analysis focused on four major EO components identified via gas chromatography-mass spectrometry (GC–MS): eugenol (cinnamon EO, CEO), carvacrol (oregano EO, OEO), thymol (thyme EO, TEO), and camphor (rosemary EO, REO). For clarity, encapsulation efficiency (%EF) is used throughout to denote the proportion of applied EO retained per 100 mg of yeast biomass.

**Fig. 4:**
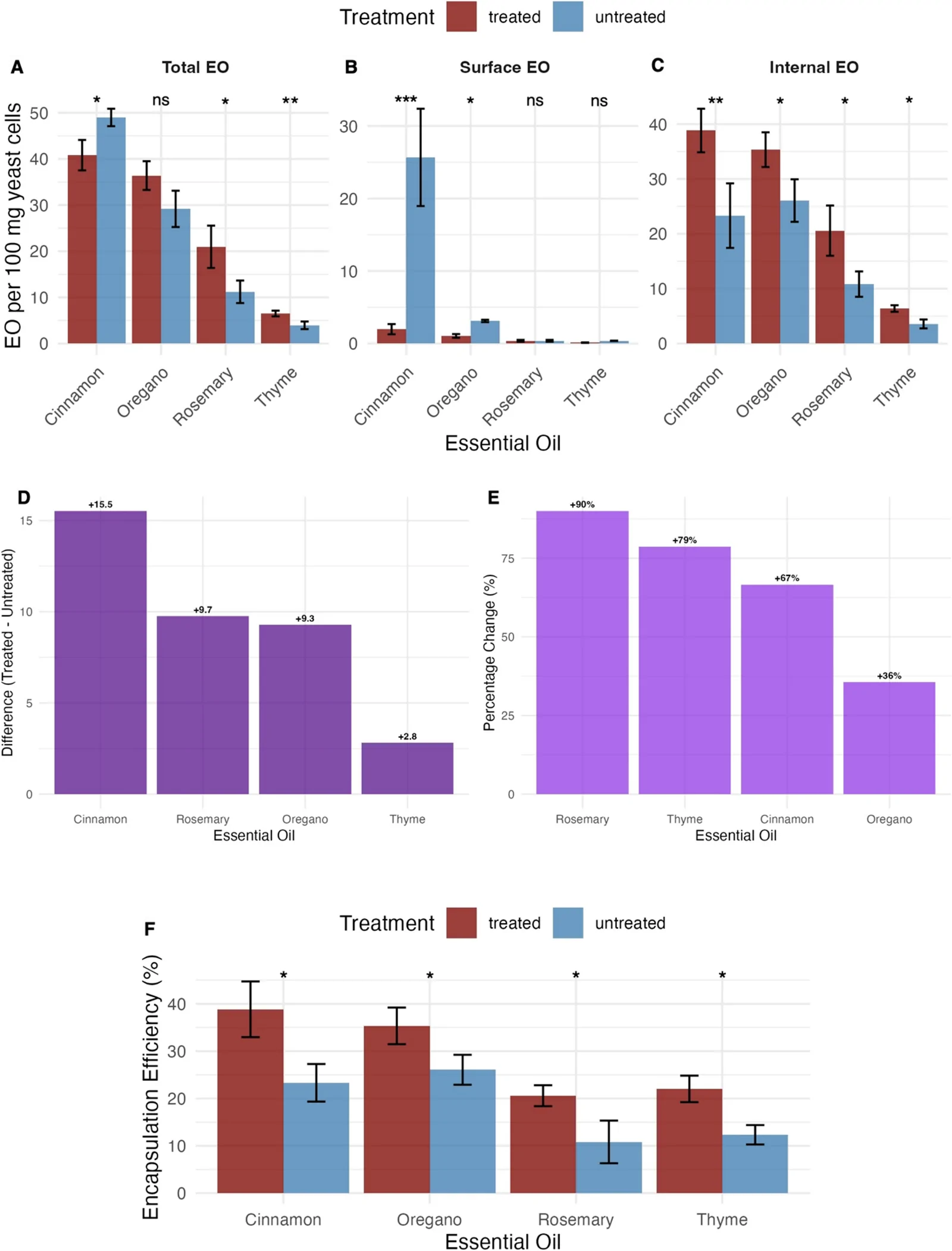
Comprehensive visualization suite of essential oil encapsulation analysis. Comparison of of (**A**) total, (**B**) surface, and (**C**)internal EO distribution in yeast cells (per 100 mg) across four essential oils under treated and untreated conditions. Treatment effect on internal EO encapsulation, (**D**) absolute difference in internal EO between treated and untreated conditions (**E**) percentage change in internal EO following treatment. Positive values indicate enhanced internal encapsulation. and (**F**) Encapsulation efficiency comparison between treated and untreated conditions, Efficiency values represent the percentage of EO retained after encapsulation relative to initial loading

As illustrated in Fig. 4A-C, treated (autolysed) yeast cells demonstrated substantially different EO %EF profiles compared to untreated (non-autolysed) cells across all four essential oils. Treated cells showed enhanced total EO %EF for thyme, rosemary, and oregano, while cinnamon exhibited higher total %EF in untreated cells. Specifically, thyme EO %EF increased from 3.95 ± 0.82 mg to 6.51 ± 0.62 mg (65% improvement), while rosemary EO %EF increased from 11.21 ± 2.44 mg to 20.96 ± 4.59 mg (87% improvement). Oregano EO %EF also improved, rising from 29.18 ± 3.93 mg to 36.39 ± 3.12 mg (24% improvement). In contrast, untreated cells exhibited significantly higher %EF of cinnamon EO (48.99 ± 1.89 mg) compared to treated cells (40.82 ± 3.30 mg), representing a 17% reduction following treatment.

Analysis of surface-bound EO (Fig. 4B) revealed pronounced differences for cinnamon EO, with untreated cells showing higher surface binding (25.67 ± 6.71 mg) compared to treated cells (1.98 ± 0.70 mg). For other EOs, surface binding was minimal in both conditions, with treated values ranging from 0.12 ± 0.03 mg (thyme) to 1.05 ± 0.26 mg (oregano). The treatment's most significant impact was observed in internal EO encapsulation (Fig. 4C). Treated cells showed significantly higher internal EO %EF across all essential oils except cinnamon, where treatment actually increased internal encapsulation despite reducing total retention. This paradoxical result suggests that the treatment may alter yeast cell wall properties in ways that preferentially enhance external binding sites for certain EO components while potentially creating exclusion mechanisms for others. This is further substantiated as shown in Fig. 4D-E, where the treatment effect varied considerably among EOs: cinnamon showed the greatest absolute improvement in internal encapsulation (+ 15.5 units), while thyme exhibited the highest percentage increase (+ 78%).

Encapsulation efficiency analysis (Fig. 4F) revealed that among the four EOs analysed, treated yeast cells showed the highest EF for CEO, reaching 38.84% ± 5.88. In contrast, REO in untreated cells displayed the lowest retention, with an EF of 10.82% ± 4.51. Oregano and thyme EOs demonstrated intermediate EF values of 35.34% ± 3.87 and 22.02% ± 2.80, respectively, in treated cells. The treatment significantly (p < 0.05) enhanced EF for all four EOs compared to untreated controls, with rosemary EO showing the most pronounced enhancement (1.90-fold increase, 90.2% improvement), followed by thyme EO (1.79-fold, 78.6% improvement), cinnamon EO (1.67-fold, 66.6% improvement), and oregano EO (1.36-fold, 35.6% improvement). These differences in loading are partly consistent with the physicochemical nature of the dominant constituents shown in Table 2: the lipophilic aromatic structures of eugenol and carvacrol favour partitioning into and retention within the yeast lipid bilayer, whereas the more volatile bicyclic ketone camphor is retained less efficiently.

### Determination of antimicrobial efficiency of yeast-encapsulated EOs

**Fig. 5:**
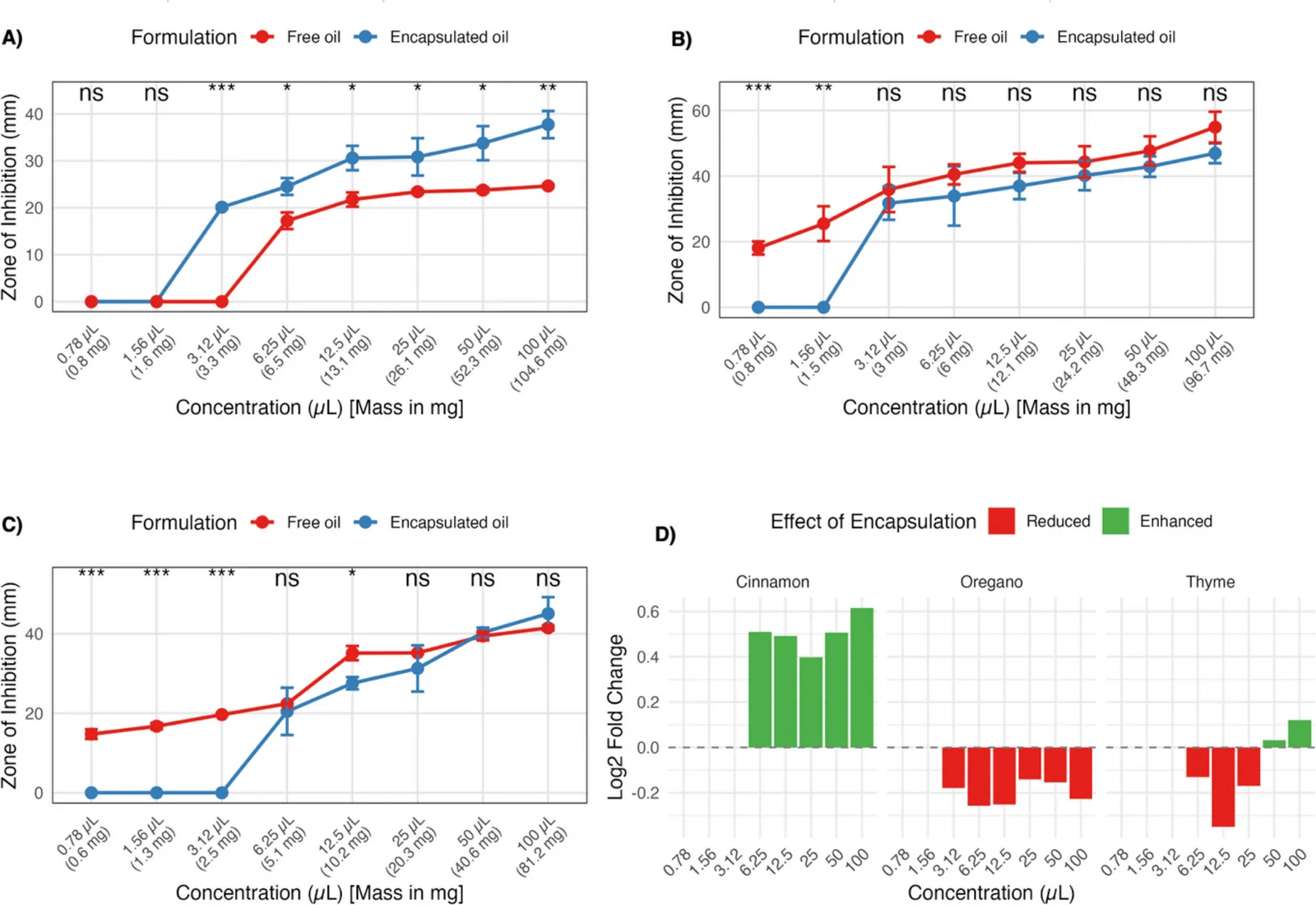
Concentration-dependent antimicrobial activity of essential oils in free and encapsulated formulations of (**A**) Thyme, (**B**) Oregano, and (**C**) Cinnamon essential oils. Inhibition zones represent the mean ± SD of three independent replicate. Statistical significance (*p < 0.05, **p < 0.01, ***p < 0.001) indicates treatment-dependent efficacy variations. (**D**) Quantitative assessment of encapsulation efficacy on antimicrobial activity. Positive values (green) indicate enhancement through encapsulation, while negative values (red) indicate reduction

The antimicrobial efficacy of three encapsulated essential oils (cinnamon, thyme, and oregano) was evaluated against *Escherichia coli*, an important food spoilage pathogen, using agar overlay assays. To ensure accurate comparisons between free and encapsulated formulations, the equivalent mass of free EO was determined for encapsulated EO in freeze-dried yeast cells using the mass equivalent of the applied volume, based on the known average mg of EO that can be encapsulated in yeast cells for each oil. The minimum inhibitory concentration analysis revealed differential susceptibility patterns between free and encapsulated formulations (Fig. 5 A, B and C). Encapsulated cinnamon essential oil demonstrated significantly higher potency compared to OEO and TEO, with its MIC reduced by 50% from 6.25 µL (6.54 mg) to 3.12 µL (3.26 mg). In contrast, encapsulation adversely affected oregano essential oil, increasing its MIC fourfold from 0.78 µL (0.75 mg) to 3.12 µL (3.02 mg). Similarly, encapsulation increased the MIC of thyme essential oil eightfold from 0.78 µL (0.63 mg) to 6.25 µL (5.08 mg), indicating that encapsulation may reduce the immediate bioavailability of oregano and thyme oils at lower concentrations.

Considering that the liquid matrix could influence the release kinetics of EO from yeast cells, a follow-up experiment on solid agar was performed to determine the differential effect of yeast encapsulation on antimicrobial efficacy under conditions more representative of potential food surface applications. The solid agar results (Figure S4 and 5D) revealed distinct patterns consistent with the liquid matrix findings but provided additional insights into concentration-dependent effects. On solid agar, Encapsulated CEO showed significantly enhanced antimicrobial activity across all concentrations from 1.3 mg (3.12 µL) to 81.2 mg (100 µL) (p < 0.05), with the most substantial improvement observed at 0.6 and 1.3 mg (p < 0.001). The encapsulated form consistently produced larger inhibition zones than the free form, with mean differences ranging from 7.31 to 20.12 mm across concentrations. For TEO, the encapsulated form produced significantly larger inhibition zones than the free form at intermediate concentrations, particularly at 12.1 mg (12.5 µL) (p < 0.05). However, at lower concentrations (0.78–3.12 µL), the free form demonstrated significantly greater activity (p < 0.001), explaining the higher MIC for encapsulated TEO. At the highest concentration (100 µL), both formulations showed comparable activity without statistical significance. The free form OF oregano essential oil showed generally higher activity than the encapsulated form, this difference reached statistical significance only at the lowest concentrations (0.78–1.56 µL, p < 0.01). At higher concentrations (3.12–100 µL), no significant differences were observed.

### Antibacterial activity of free and encapsulated EOs in food models

**Fig. 6:**
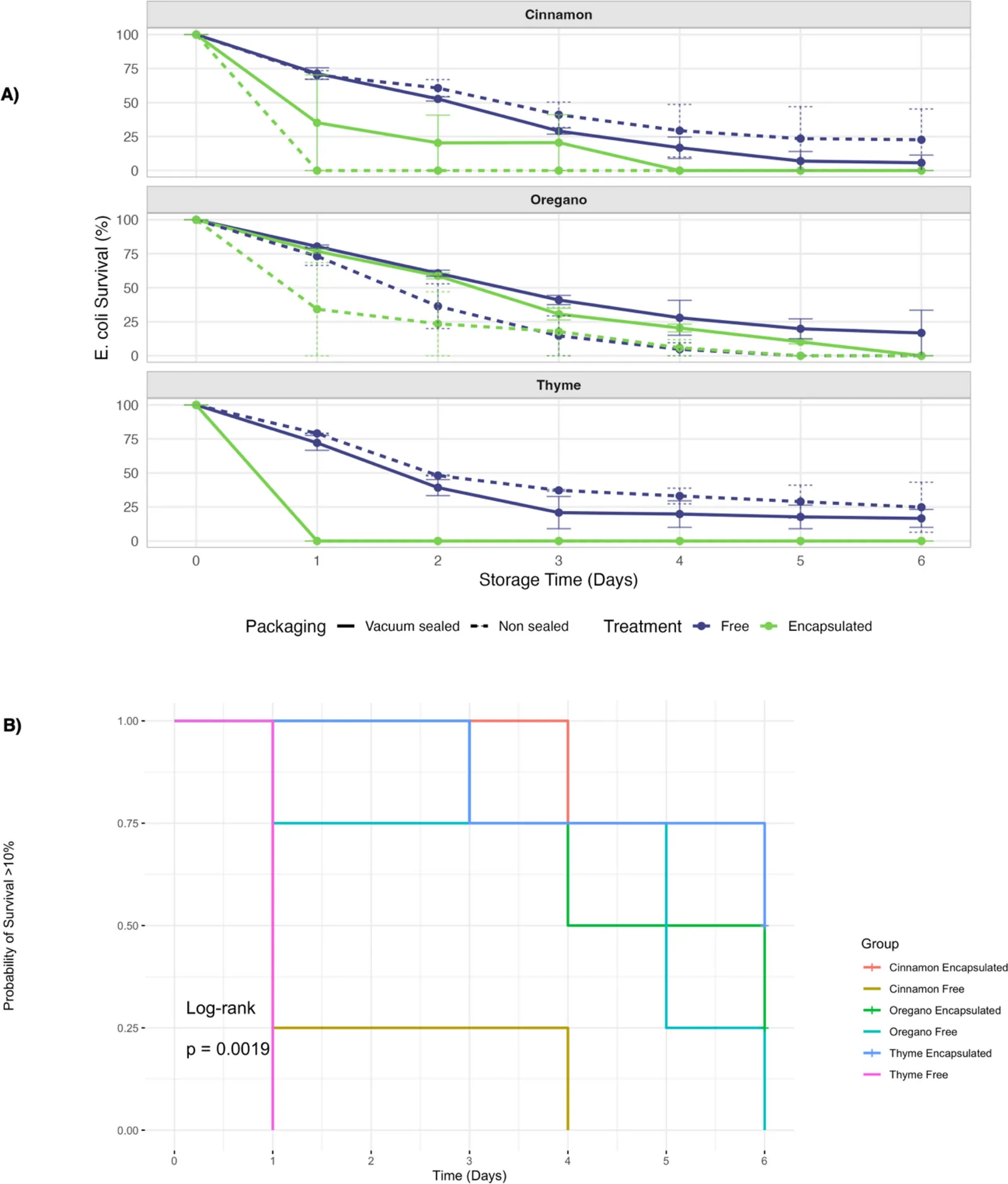
(**A**) Time-course survival curves for Cinnamon, Oregano, and Thyme EOs under different packaging and treatment conditions (**B**) Kaplan–Meier survival curves showing time to < 10% E. coli survival by oil type and treatment

This study evaluated the antimicrobial effectiveness of free and yeast-encapsulated essential oils against *E. coli* in preservative-free ham over a six-day storage period. The extended observation window beyond typical two-day shelf life provided proof-of-concept data on short-term antimicrobial effects, establishing baseline information for future studies on long-term microbial stability and product quality. Mixed-effects model analysis revealed significant main effects of essential oil type (F (2,143) = 6.25, p = 0.0025), treatment type (F (1,143) = 27.41, p = 5.77 × 10⁻⁷), and storage time (F (1,143) = 167.19, p = 8.13 × 10⁻^2^⁶) on *E. coli* survival (Table S1). Significant interactions were observed between oil type and treatment (p = 0.026) and between oil type and time (p = 0.034). Specifically, encapsulated thyme EO demonstrated immediate and complete bactericidal action, eliminating *E. coli* by Day 1 in both vacuum-sealed (0% survival) and non-sealed conditions (0% survival). In contrast, free thyme EO showed gradual decline, with vacuum-sealed samples reaching 16.6% survival by Day 6 and non-sealed samples reaching 24.8% survival (Fig. 6A). The exponential decay model revealed a half-life of 1.68 days for free thyme under vacuum-sealed conditions versus 0.014 days for encapsulated forms (Table S2). Encapsulated oregano EO showed enhanced but more gradual antimicrobial action. Under vacuum-sealed conditions, *E. coli* survival declined to 0% by Day 6, while non-sealed conditions showed 34.3% survival on Day 1, declining to 0% by Day 5. Free oregano EO exhibited slower reduction, with vacuum-sealed samples maintaining 16.8% survival at Day 6 and non-sealed samples reaching 0% survival by Day 5 (Fig. 2B). The decay model indicated half-lives of 2.29 days (free, vacuum) versus 0.86 days (encapsulated, non-sealed). Encapsulated cinnamon EO demonstrated rapid antimicrobial effects in non-sealed conditions (0% survival by Day 1) and slightly delayed action in vacuum-sealed conditions (0% survival by Day 4). Free cinnamon EO showed moderate reduction, with non-sealed samples maintaining 22.7% survival at Day 6 and vacuum-sealed samples reaching 5.7% survival (Fig. 6A). Decay analysis revealed half-lives of 1.68 days (free, vacuum) versus 0.01 days (encapsulated, non-sealed) (Table S2).

Kaplan–Meier analysis evaluated time-to-event (survival < 10%) without packaging as a factor (Fig. 6B). Median survival times revealed significant differences where encapsulated treatments reached the event threshold significantly faster than free treatments. For cinnamon, median survival time decreased from 4.5 days (free) to 1 day (encapsulated); for oregano, from 3.5 days to 3 days; and for thyme, from 3 days to 1 day. Log-rank test confirmed significant differences among groups (χ^2^ = 35.2, p < 0.001), indicating that both oil type and encapsulation status significantly affected *E. coli* survival dynamics.

Post-hoc comparisons (Tukey-adjusted) identified significant pairwise differences (Table 2). The most effective treatment combinations were encapsulated cinnamon in non-sealed conditions and encapsulated thyme in both packaging conditions, which showed significantly lower survival compared to all free EO treatments (p < 0.001). Encapsulated oregano in vacuum-sealed conditions was significantly more effective than free oregano in vacuum-sealed conditions (p = 0.026).

### Physicochemical quality of yeast treated ham

The physicochemical quality of the product is likely to be a key concern for both producers and consumers, so these parameters were measured in the treated ham after six days of storage. The results (Table 3) compare the colour and texture of untreated ham with that of ham treated with encapsulated EO. Water activity was essentially unchanged by the treatment (0.88 in untreated versus 0.89 in treated ham; no significant difference), indicating that the encapsulated EO did not alter the moisture status of the product. Maximum shear force, an index of firmness, was higher in the treated ham (59.62) than in the untreated ham (50.16), but this difference was not statistically significant, so texture was largely preserved. Colour was more affected: the treated ham was significantly darker (lower L*, 51.19 versus 59.71) and significantly redder (higher a*, 8.05 versus 5.76), whereas the increase in yellowness (b*, 23.00 versus 21.33) was not significant.

**Table 3:** Average water Activity (aw), texture and colour of raw untreated ham and of ham treated with encapsulated EO after six days of storage

Parameters	Untreated raw ham	EO-treated ham
Water activity	0.88^a^ ± 0.01	0.89^a^ ± 0.10
Maximum Shear Force	50.16^a^ ± 9.08	59.62^a^ ± 8.14
L*	59.71^a^ ± 2.20	51.19^b^ ± 0.50
a*	5.76^a^ ± 0.77	8.050^b^ ± 0.39
b*	21.33^a^ ± 0.97	23.00^a^ ± 0.45

Values are means ± standard deviation. Within each **row**, means sharing the same superscript letter are not significantly different (p < 0.05). L* denotes lightness; a* the red–green axis (higher = redder); b* the yellow–blue axis (higher = more yellow)

## Discussion

The search for natural, effective food preservation solutions has led to renewed interest in EOs potent antimicrobial agents that face formidable challenges of volatility and instability. This study demonstrates that yeast encapsulation not only overcomes these limitations but enhances the antibacterial activity of thyme, oregano, and cinnamon EOs against *E. coli* in a ham matrix. By transforming these volatile compounds into sustained-release antimicrobial agents, we bridge the gap between laboratory efficacy and practical food preservation, offering a promising natural alternative to synthetic preservatives.

The potent antimicrobial activity of thyme, oregano, and cinnamon essential oils in this study aligns with extensive literature documenting their effectiveness against foodborne pathogens. For example, (Mollea et al. 2022) who investigated the antimicrobial efficacy of thyme and oregano EOs against *Staphylococcus epidermidis* and *E. coli* over extended periods. Their disk diffusion tests showed strong antimicrobial activity, with inhibition zones of 50 mm for thyme and 40 mm for oregano after 24 h. However, a reduction in inhibition was observed after 48 h due to evaporation, emphasising the volatility challenge that encapsulation aims to address. (Mutlu-ingok et al. 2021) reported inhibition zones of 27.8 mm and 25.3 mm for thyme and oregano EOs, respectively, against *E. coli* using 20 µL of oil. (Terrazas-pineda et al. 2025) found zones of 8.33 mm and 6.27 mm for thyme and cinnamon EOs (10 µL) against the same pathogen, while (Oulkheir et al. 2017) reported zones of 28 mm and 18 mm for thyme and cinnamon EOs (10 µL). For cinnamon EO, (Iseppi et al. 2024) reported broad-spectrum antibacterial activity, producing inhibition zones of 27–32 mm against several pathogens including *E. coli*. The inhibition zones measured in our work fall within the ranges reported in these studies.

The pronounced antimicrobial properties exhibited by the EOs can be largely attributed to their predominant bioactive phenolic constituents. GC–MS analysis confirmed that the tested oregano, thyme, and cinnamon EOs were rich in carvacrol, thymol, and eugenol, respectively. These phenolic compounds exert antimicrobial effects through multiple mechanisms, including membrane disruption, ATP depletion, and protein denaturation (Tiwari et al. 2009). The overall bioactivity of essential oils is further broadened by monoterpenes and sesquiterpenes, including α-phellandrene, α-pinene, camphene, β-myrcene, D-limonene, γ-terpinene, and caryophyllene. These compounds collectively contribute not only to antimicrobial activity but also to distinctive aromatic profiles, anti-inflammatory, and antioxidant properties (Holzke et al. 2006; Jayaprakasha and Rao 2011; Do Nascimento et al. 2020; Masyita et al. 2022; Câmara et al. 2024; Siddiqui et al. 2024). Among phenolics, thymol and carvacrol are particularly recognized for their strong antimicrobial, antifungal, and antioxidant activities (Do Nascimento et al. 2020; Soleimani et al. 2022). Eugenol, a phenylpropanoid, exhibits a versatile pharmacological profile encompassing antimicrobial, anti-inflammatory, analgesic, neuroprotective, antidiabetic, and antitumor activities, highlighting its potential in preventing and treating various disorders (Nisar et al. 2021). Beyond these, other terpenoids such as camphor and oxygenated compounds like eucalyptol (1,8-cineole) also demonstrate significant antifungal potential (Preljević et al. 2024). The structural features underlying this activity are evident from the constituent structures in Table 2: carvacrol and thymol are isomeric monoterpene phenols bearing a hydroxyl group on an aromatic ring, a feature widely associated with disruption of microbial membranes, whereas eugenol is a phenylpropanoid carrying both a phenolic hydroxyl and an allyl side chain. By contrast, the main rosemary constituents camphor and eucalyptol lack a free phenolic hydroxyl, consistent with the comparatively weaker antibacterial activity of rosemary oil observed here.

Encapsulation temperature is a critical factor influencing performance. Typically, temperatures between 20 °C and 60 °C are used, with around 40 °C considered ideal because it enhances membrane fluidity and improves compound permeation (Bishop et al. 1998). In this study, the optimal temperature for loading EO into *S. cerevisiae* was 30—45 °C, determined based on three parameters: cell integrity, cell viability, and oil retention capacity. This result aligns partly with the findings of (Dimopoulos et al. 2021), who investigated the effects of different temperatures (30 °C, 37 °C, 45 °C, and 65 °C) and reported no significant differences in encapsulation efficiency at 30–45 °C, but a sharp decrease at 65 °C, consistent with our observation that excessively high temperatures impair the process.

In our experiments, yeast cells exposed to 62 °C showed complete loss of yeast cell reproduction with no visible colonies observed on PDA, indicating irreversible thermal damage to yeast cell structure and possibly reproductive function. The survival patterns observed support Arthur and Watson (1976), who noted that yeast cells remain viable at moderate thermal conditions (37—45 °C) while maintaining their capacity to encapsulate EO compounds. In contrast to our finding, (Nakhaee Moghadam et al. 2021) reported significantly higher loading efficiency for *Zataria multiflora* essential oil in yeast at 40–50 °C compared to 30 °C. They suggested that temperatures slightly above the membrane phase transition threshold enhance compound permeation, facilitating bioactive diffusion into the yeast cells. This difference can be explained by membrane physicochemical states, above approximately 35 °C, phospholipids adopt a liquid-crystalline state that increases membrane flexibility and promotes molecular diffusion, thereby improving encapsulation effectiveness. Conversely, below 35 °C, phospholipids transition to a gel-like state, reducing fluidity and creating a barrier that can restrict compound penetration, potentially lowering encapsulation efficiency and bioactive availability (Paramera et al. 2014).

Our results indicate that yeast encapsulation can differentially modulate the efficacy of individual EOs. As illustrated in Fig. 5, encapsulated cinnamon EO demonstrated a more substantial increase in antibacterial activity compared to its free form than was observed for thyme or oregano EOs. This suggests encapsulation may offer particular advantages for EOs like CEO, potentially by enhancing the stability and providing a gradual release of its key volatile compound, eugenol, thereby prolonging its effective antimicrobial concentration at the target site. Currently there is scarcity of comparable data on antimicrobial efficacy of yeast-encapsulated essential oil, however, these encapsulation findings are consistent with previous studies utilizing different carrier systems. Esmaeili and Asgari (2015) reported enhanced antibacterial activity of *Carum copticum* EO against *S. aureus* and *E. coli*, following encapsulation with chitosan nanoparticles. The *in-situ* analysis of encapsulated EO using raw ham demonstrated that yeast encapsulation transforms thyme, oregano, and cinnamon EOs into effective antimicrobial agents for meat preservation. The application of encapsulated EOs at their MIC resulted in a sharp, significant reduction in *E. coli* populations in ham over a six-day storage period, outperforming both free EO treatments and control samples across all tested packaging conditions. We attributed this enhanced efficacy to the encapsulation technology, which protects the volatile bioactive compounds and facilitates a gradual, sustained release at the food surface, thereby maintaining a lethal concentration for a prolonged duration. This interesting finding serves as a proof-of-concept of the potential of encapsulated EOs as a promising natural alternative to synthetic preservatives, aligning with the urgent need to combat antimicrobial resistance (AMR) in the food chain through natural, multi-target antimicrobials. While the principle of encapsulation to enhance EO efficacy is well-established using various synthetic or food-grade carriers, the novelty of this work lies in its utilization of *S. cerevisiae* as a bio-preservative carrier. This yeast-based system offers distinct potential advantages, including the use of a GRAS organism, the ability to protect and control the release of bioactive compounds, and the possibility of synergistic effects from the yeast cell wall components themselves. Because formal release-kinetics were not measured in this study, references to controlled or sustained release are made cautiously and are inferred from the prolonged bactericidal effect observed and from the water-activity-dependent release previously reported for this carrier system (Dimopoulos et al. 2021).

The broader literature consistently supports that encapsulation, regardless of the carrier material, fundamentally improves the practical antimicrobial application of EOs. For instance, research on other encapsulation systems in meat matrices aligns with our findings of enhanced efficacy. (Hadian et al. 2017) investigated the antimicrobial effects of free and nano-encapsulated rosemary essential oil coatings with chitosan (CS)-benzoic acid (BA) nanogel against *Salmonella typhimurium* on beef cutlet samples, evaluating their efficacy over 12 days of refrigerated storage at 4 °C. The results showed that in the control samples, the *S. typhimurium* population significantly increased from 9.3 to 10.38 log CFU/g over the storage period. (Labarca 2017) evaluated the effect of cinnamon bark essential-oil microcapsules, using maltodextrin and gum Arabic as coating materials. Their results showed that the addition of cinnamon bark essential-oil microcapsules inhibited microbial growth in the ground beef, with higher microcapsule concentrations producing greater inhibitory effects. Furthermore, the findings of (Gottardo et al. 2022) on the anti-*Listeria* activity of free and encapsulated EOs in Italian salami demonstrated that the reduction of *Listeria monocytogenes* in the meat matrix increased as the concentration of encapsulated EO increased. Their study recorded an inhibition of *L. monocytogenes* at a minimum inhibitory concentration (MIC) of 0.3% (3 mg/mL). These studies confirm that encapsulation provides a significant antimicrobial hurdle in solid, complex food environments. However, the outcomes can be matrix- and system-dependent, highlighting important considerations for application. The work of Nakhaee et al. (2020**)** illustrated that in a homogeneous liquid matrix like chicken soup, the antibacterial activity of free and encapsulated *Zataria multiflora* EO against *E. coli* and *L. monocytogenes* was not significantly different over 14 days. This contrasts with solid meat results and suggests that in systems where EO dispersion is not a limiting factor, the relative advantage of encapsulation may shift more toward long-term stability rather than immediate bioavailability. Furthermore, Gottardo et al. (2022) provided a critical practical insight: while the in vitro MIC for their encapsulated EO blend against *Listeria monocytogenes* was 0.3%, a higher concentration was required to achieve the same inhibition in Italian salami. From a scale-up perspective, both the autolysis and encapsulation steps use inexpensive, food-grade *S. cerevisiae* biomass and conventional unit operations (incubation, centrifugation, and drying) already established at industrial scale in the baker’s-yeast and yeast-extract industries. This strongly supports reasonable technical feasibility; however, the additional autolysis, loading, and washing steps add processing cost, and a formal techno-economic comparison with established routes such as spray-drying and coacervation was beyond the scope of this proof-of-concept study.

## Conclusion

This study systematically evaluated the antimicrobial potential of nine EOs (angelica root, clove bud, cinnamon leaf, rosemary, sage, lemongrass, oregano, ginger, and thyme) against a panel of microbial targets, comprising the bacteria *E. coli* and *E. faecium* and the fungi *A. niger* and *P. aerogenes*. Based on initial screening via the agar diffusion method, three of the most potent EOs (oregano, thyme, and cinnamon) were selected for microencapsulation within *S. cerevisiae* cells, a GRAS and biodegradable carrier. Our findings demonstrate that yeast-based microencapsulation is a promising strategy for overcoming the intrinsic limitations of EOs, such as high volatility, susceptibility to oxidation, and poor aqueous solubility. A pre-encapsulation autolysis treatment substantially increased the oil-absorption capacity of the cells and improved encapsulation efficiency, and the encapsulated oils retained, and in some cases enhanced, their antimicrobial activity relative to the free oils.

In a ham model system, yeast-encapsulated formulations of thyme, oregano, and cinnamon EOs achieved a sustained and significant reduction of *E. coli* populations over a six-day storage period, outperforming their free oil counterparts and confirming the technology's utility in a complex food matrix. However, the antimicrobial validation was conducted primarily against *E. coli* in a single meat product only. The effectiveness of this encapsulation system against other pathogens (especially fungi like *A. niger*) and in diverse food matrices (e.g., dairy, produce, high-fat products) remains unknown and warrants investigation. The impact of the encapsulated oils on the sensory properties of the food (flavour, aroma, and color) was not evaluated, and the economic and technical feasibility of scaling up the autolysis and encapsulation steps for industrial production has yet to be examined.

Overall, this work provides proof-of-concept that yeast microencapsulation is a promising, sustainable approach for improving the delivery and performance of natural antimicrobial EO in meat. By protecting bioactive EO compounds, this method holds significant potential for future applications ranging from natural food preservation to pharmaceutical formulations. Realising this potential will require further validation across a wider range of target organisms, food matrices, and storage conditions, together with sensory and scale-up assessments.

## Supplementary Information

Below is the link to the electronic supplementary material.

Supplementary file1 (DOCX 1737 KB)

## Data Availability

All data supporting the findings of this study are available within the paper and its supplementary information.
