# Supplementary material for: Enhancing meat preservation with eco-friendly, antibiotic-free yeast-encapsulated essential oils
Source: World J Microbiol Biotechnol. 2026 Aug 27;42(9):489. doi: 10.1007/s11274-026-05213-4 (PMC13522087; doi:10.1007/s11274-026-05213-4)
Supplement: Supplementary file 1 [file 11274_2026_5213_MOESM1_ESM.docx]

Supplementary files

Figure S1: *Polynomial A) Eugenol B)* *Carvacrol C) Thymol and D)* *Camphor GC-MS Calibration Curve using peak area at m/z 164, 150, 135 and 152, respectively.*

Figure S3: A) Phenotypic and B) microscopic confirmation of yeast cell viability after encapsulation with essential oil

Figure S2: Representative gas chromatography-mass spectrometry (GC-MS) chromatograms of the four essential oils selected for encapsulation. The peak corresponding to the predominant bioactive compound in each oil is labeled: **(a)**Cinnamon leaf oil (predominant compound: eugenol), **(b)** Oregano oil (predominant compound: carvacrol), **(c)** Thyme oil (predominant compound: thymol), and **(d)**Rosemary oil (predominant compound: camphor).

Figure S4: Comparative agar diffusion assay showing the antimicrobial activity of essential oils (EOs) and their primary constituents against *Escherichia coli* at a standard dose of 100 µL. Each column represents a different EO: (A) oregano (OEO), (B) thyme (TEO), and (C) cinnamon (CEO). For each oil, the activity of the yeast-encapsulated form (Row 1), the free oil (Row 2), and its purified main bioactive compound (Row 3: carvacrol, thymol, and eugenol, respectively) is displayed.

Table S1: ANOVA results from mixed-effects model analysing the effects of essential oil type, packaging condition, treatment (free vs. encapsulated), storage time, and their interactions on *E. coli* survival in preservative-free ham.

|  | Sum Sq | Mean Sq | NumDF | DenDF | F value | Pr(>F) |
| --- | --- | --- | --- | --- | --- | --- |
| Oil | 2.83703405 | 1.41851702 | 2 | 143 | 6.25161846 | 0.0024955 |
| Packaging | 0.56672396 | 0.56672396 | 1 | 143 | 2.49763794 | 0.11622658 |
| Treatment | 6.21907259 | 6.21907259 | 1 | 143 | 27.4083908 | 5.77E-07 |
| day | 37.9363917 | 37.9363917 | 1 | 143 | 167.1914 | 8.13E-26 |
| Oil:Packaging | 0.38451544 | 0.19225772 | 2 | 143 | 0.84730877 | 0.43070685 |
| Oil:Treatment | 1.69255604 | 0.84627802 | 2 | 143 | 3.72967486 | 0.02636667 |
| Packaging:Treatment | 0.61902111 | 0.61902111 | 1 | 143 | 2.72811938 | 0.10078958 |
| Oil:day | 1.57122415 | 0.78561207 | 2 | 143 | 3.4623109 | 0.03401026 |
| Packaging:day | 0.00233261 | 0.00233261 | 1 | 143 | 0.01028015 | 0.91938204 |
| Treatment:day | 0.2277305 | 0.2277305 | 1 | 143 | 1.00364267 | 0.3181229 |
| Oil:Packaging:Treatment | 0.22012213 | 0.11006107 | 2 | 143 | 0.48505571 | 0.61667212 |
| Oil:Packaging:day | 0.83937384 | 0.41968692 | 2 | 143 | 1.84962355 | 0.16103968 |
| Oil:Treatment:day | 0.19521366 | 0.09760683 | 2 | 143 | 0.43016802 | 0.65123862 |
| Packaging:Treatment:day | 0.21252916 | 0.21252916 | 1 | 143 | 0.93664805 | 0.33477536 |
| Oil:Packaging:Treatment:day | 0.35163087 | 0.17581543 | 2 | 143 | 0.77484513 | 0.46269984 |

**Table S2. Exponential Decay Model Parameters for *E. coli* Survival**

| **Oil** | **Packaging** | **Treatment** | **Initial Value (a)** | **Decay Rate (b)** | **Half-life (days)** | **R²** |
| --- | --- | --- | --- | --- | --- | --- |
| Cinnamon | Vacuum | Free | 103.35 | 0.41 | 1.68 | 0.96 |
| Cinnamon | Vacuum | Encapsulated | 98.07 | 0.84 | 0.83 | 0.76 |
| Cinnamon | Non-sealed | Free | 98.44 | 0.28 | 2.48 | 0.75 |
| Cinnamon | Non-sealed | Encapsulated | 100.00 | 49.68 | 0.01 | 1.00 |
| Oregano | Vacuum | Free | 103.43 | 0.30 | 2.29 | 0.91 |
| Oregano | Vacuum | Encapsulated | 105.57 | 0.39 | 1.78 | 0.96 |
| Oregano | Non-sealed | Free | 105.69 | 0.56 | 1.24 | 0.92 |
| Oregano | Non-sealed | Encapsulated | 97.48 | 0.80 | 0.86 | 0.76 |
| Thyme | Vacuum | Free | 100.63 | 0.41 | 1.68 | 0.91 |
| Thyme | Vacuum | Encapsulated | 100.00 | 49.68 | 0.01 | 1.00 |
| Thyme | Non-sealed | Free | 98.64 | 0.28 | 2.49 | 0.87 |
| Thyme | Non-sealed | Encapsulated | 100.00 | 49.68 | 0.01 | 1.00 |

**Table S3. Key Significant Pairwise Comparisons (Tukey-adjusted) in the ham model analysis**

| **Comparison** | **Mean Difference** | **p-value** | **Significance** |
| --- | --- | --- | --- |
| Oregano Vacuum Free vs Oregano Non-sealed Free | 1.176 | 0.020 | * |
| Oregano Vacuum Free vs Cinnamon Vacuum Encapsulated | 1.252 | 0.009 | ** |
| Oregano Vacuum Free vs Thyme Vacuum Encapsulated | 1.330 | 0.004 | ** |
| Thyme Vacuum Free vs Thyme Vacuum Encapsulated | 1.419 | 0.001 | ** |
| Thyme Non-sealed Free vs Thyme Non-sealed Encapsulated | 1.629 | <0.001 | *** |

*Note: *p<0.05, **p<0.01, ***p<0.001*
